# Using a mobile health application to support self-management in chronic obstructive pulmonary disease: a six-month cohort study

**DOI:** 10.1186/s12911-015-0171-5

**Published:** 2015-06-18

**Authors:** Maxine Hardinge, Heather Rutter, Carmelo Velardo, Syed Ahmar Shah, Veronika Williams, Lionel Tarassenko, Andrew Farmer

**Affiliations:** Oxford University Hospitals NHS Trust, UK, Oxford; Oxford Health Foundation Trust, UK, Oxford; Department of Engineering Science, University of Oxford, UK, Oxford; Nuffield Department of Primary Care Health Sciences, University of Oxford, UK, Oxford

**Keywords:** Chronic obstructive pulmonary disease, Chronic condition, Self-management, Telehealth, Digital health, E-health, Mobile health, Alerts

## Abstract

**Background:**

Self-management strategies have the potential to support patients with chronic obstructive pulmonary disease (COPD). Telehealth interventions may have a role in delivering this support along with the opportunity to monitor symptoms and physiological variables. This paper reports findings from a six-month, clinical, cohort study of COPD patients’ use of a mobile telehealth based (mHealth) application and how individually determined alerts in oxygen saturation levels, pulse rate and symptoms scores related to patient self-initiated treatment for exacerbations.

**Methods:**

The development of the mHealth intervention involved a patient focus group and multidisciplinary team of researchers, engineers and clinicians. Individual data thresholds to set alerts were determined, and the relationship to exacerbations, defined by the initiation of stand-by medications, was measured. The sample comprised 18 patients (age range of 50–85 years) with varied levels of computer skills.

**Results:**

Patients identified no difficulties in using the mHealth application and used all functions available. 40 % of exacerbations had an alert signal during the three days prior to a patient starting medication. Patients were able to use the mHealth application to support self- management, including monitoring of clinical data. Within three months, 95 % of symptom reporting sessions were completed in less than 100 s.

**Conclusions:**

Home based, unassisted, daily use of the mHealth platform is feasible and acceptable to people with COPD for reporting daily symptoms and medicine use, and to measure physiological variables such as pulse rate and oxygen saturation. These findings provide evidence for integrating telehealth interventions with clinical care pathways to support self-management in COPD.

## Background

Chronic obstructive pulmonary disease (COPD) is a common condition worldwide which has a significant impact on all domains of patients’ health related quality of life [1, 2]. In addition, one in eight UK acute emergency admissions is the result of an acute COPD exacerbation, and 90 day re-admission rates are as high as 30 % [3].

Self-management strategies have the potential to improve quality of life and reduce hospital admissions [4]. However results of existing studies employing a variety of self-management strategies are mixed [5, 6] and it remains unclear which forms of self-management are suitable for individual patients, or how they are best placed in patient pathways and supported by community respiratory nurses in conjunction with primary care teams. Therefore innovative approaches to self-management are required, which maintain a patient-centred approach.

The use of telehealth interventions has the potential to help patients self-manage their condition by delivering individually tailored education and treatment plans, and by providing support to patients to monitor and interpret their own physiological data. In addition, these interventions can reduce patients’ sense of isolation and improve self-efficacy [7]. Telehealth also has the potential to allow health care professionals to monitor remotely for deteriorations or long-term trends, and offers opportunities for intervention to improve outcomes. Systematic reviews of the use of telehealth in COPD have focused on telemonitoring with remote decision-making by health care professionals and have not shown convincing evidence of effectiveness on hospital admissions [8]. Despite the lack of evidence there are many telehealth projects currently underway in a variety of NHS organisations which remain poorly validated.

The specifications for a telehealth solution that could be implemented in NHS practice, and are likely to be feasible and acceptable to both patients and health care professionals would include low cost, ease-of-use, based on a generic technology and integrated within care pathways. In addition, it would need to be capable of deployment on a large scale and of delivering improved self-management.

The sElf-management anD support proGrammE (EDGE) project [9], was set up to develop and test the efficacy of an internet-linked tablet computer based mobile health (mHealth) system, designed according to these specifications, in improving quality of life in patients with moderate to severe COPD. The mHealth system was developed for use by people with varying computer skills, with the aim of ensuring data quality and integration of the system within clinical care to deliver self-management support. We set out to evaluate the use of the system in a cohort study during which directly-measured clinical variables and patient-reported variables were measured throughout a six-month period.

## Methods

### Development of mHealth system

The mHealth system used in this study consisted of an internet-linked Android tablet computing device running a software application, which was intended for use by people with varying computer skills. A multi-disciplinary team was involved in the design of the system and the implementation of the project: this included primary care physicians, respiratory nurses, a secondary care respiratory physician, a psychiatrist, and engineers. A small group of patients, who were not involved in the cohort study, acted as a participant informant group and provided guidance and feedback for development of the software application. The involvement of a patient focus group enabled the software to be optimised so that patients could record data with ease and access educational materials (an example of user interface can be seen in the Appendix). Evidence-based self-management strategies were included in the education materials which were based on the principles of pulmonary rehabilitation. Technical development focused on enabling the acquisition of high-quality data, with artefactual data filtered out, and on making the data visible in real time to both patient users and healthcare professionals. The resulting mHealth system was integrated within existing local clinical care pathways following discussion with patients about how they might use data, and with relevant healthcare professionals about how they would monitor data and interact with patients (an overview of the system and interactions with patients and health professionals is available in the Appendix).

The self-management module of the mHealth system included the facility for patients to keep a daily symptom diary which included questions about their general well-being, cough and sputum production (quantity and colour), and breathlessness to allow exacerbation detection based on questions used in previous trials [10, 11]. An option to record medication use was included with diary-based questions on use of inhalers, antibiotics and oral steroids (or a combination of these medicines). An exacerbation ‘event’ was deemed to have occurred when patients initiated the use of either antibiotics, oral steroids or both antibiotics and steroids. Pulse rate and oxygen saturation, computed from a 30-s period of data acquired with a pulse oximeter (Nonin Onyx II 9650) and its finger probe, were collected daily and transmitted wirelessly to the tablet (Samsung Galaxy Tab 2) via Bluetooth. Additional self-management support was provided via software modules: (i) personalised plans for self-management developed from a standard care plan and for treating an exacerbation; (ii) 14 brief video clips and text-based material (see Table 1) providing disease education, advice on managing COPD including diet, medicine use and inhaler technique (patients could only view the inhalers they had been prescribed); (iii) the facility to receive a brief message from the trial respiratory nurse. In addition, personalised self-management plans could be modified or updated remotely according to patients’ needs. The videos were scripted by a respiratory research nurse, reviewed by the respiratory consultant and filmed by the research team. In addition, personalised self-management plans and inhalers videos could be modified or updated remotely as patients required.

**Table 1:** Video clips to support self-management and frequency of viewing

	Video ID	Video Title
Inhaler technique videos	1	How to use the Handihaler
	2	How to use the Accuhaler
	3	How to use the Metered Dose Inhaler
	4	How to use the Turbohaler
	7	How to use the Aerochamber
	11	How to use the Respimat
	14	How to use the Volumatic
Exercise and pulmonary rehabilitation	5	Pulmonary rehabilitation importance
	6	Stay active
	9	Manage my breathing
	13	My breathing exercises
Mood management	8	Manage my mood
	10	Manage my worry
	12	Relaxation technique

The patient-acquired data, after completion of the diary on the tablet computer, were automatically synchronised with a remote, secure server (within the NHS network and behind the NHS firewall). Data collected were analysed using patient-specific probabilistic models. For each patient, an initial 6-week (training) period was used to generate personalised alert thresholds for symptom score, oxygen saturation, and pulse rate. Using these thresholds, we tested all data points outside the training period and triggered an alarm whenever a data point was over (or below in case of oxygen saturation) the threshold value.

### Research ethics committee

Ethical approval was obtained from South Central Berkshire Research Ethics Committee (ref 12/SC/0437) and Research Governance was granted by Oxford Health NHS Foundation Trust and Oxford University Hospitals NHS Trust.

### Patient recruitment

For the six-month cohort study, patients meeting the inclusion criteria were identified and recruited from a variety of healthcare settings: hospital admission records, respiratory out-patient clinics, pulmonary rehabilitation programmes. Patients with COPD were also recruited through disease registers held by primary care based physicians (general practitioners) providing community based management. Inclusion criteria included aged over 40 years, a smoking pack year history of greater than 10 pack years, MRC dyspnoea scale greater than or equal to 2, post bronchodilator forced expiratory volume in one second (FEV1) of less than 80 % and an FEV1/forced vital capacity ratio of less than 70 % (which were measured pre and post study by a trained respiratory research nurse), exacerbation of COPD requiring treatment or referred to pulmonary rehabilitation within last year, absence of other significant lung disease or heart failure, life expectancy of greater than three months and ability to understand written English. Within one week of recruitment to the pilot cohort study the respiratory research nurse delivered the tablet computer during a home visit, during which full written consent was taken and the patient was instructed in the use of the tablet computer, including how to complete the symptom diary, access educational materials and use the oximetry probe. Each patient was provided with an instruction leaflet designed by the research team, and a contact number for the respiratory research nurse if they encountered any problems. Patients were asked to complete diaries and measure their oxygen saturation levels and pulse rate on a daily basis at a time of their choosing. They were informed that the data they sent would be looked at on a weekly basis to review trends: however, if they felt unwell they were to contact their usual health care professional for advice. The respiratory research nurse would contact the patients by phone in the case of a period of missing data (>7 days) or if the patient entered the alert zone for two consecutive days.

### Data collection

The time taken for the first home visit by the respiratory nurse, during which instruction on how to use the mHealth tablet was given, was recorded. Patient-reported data for symptoms and the pulse oximetry data (oxygen saturation and pulse rate) were downloaded from the study server behind the NHS firewall onto an Excel spreadsheet. The number of times patients completed self-management diaries, accessed self-management plans, educational videos and respiratory nurse messaging was also downloaded onto the Excel spreadsheet. The times taken for patients to complete their daily symptom and medication diary and their pulse rate/oxygen saturation measurements using pulse oximetry were measured. Data were reported using descriptive statistics.

### Data analysis

**Fig. 1:**
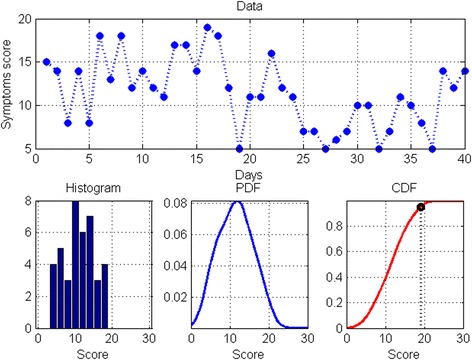
Computation of an alert threshold. Symptom score as recorded in patient’s computer tablet (top trace), histogram depicting data distribution (lower left plot), probability density function PDF (lower middle plot), cumulative density function CDF (lower right plot), with patient-specific alert threshold value derived from 95th centile of CDF

Data collected on the secure server were analysed using patient-specific probabilistic models in order to compute an alert threshold for each data type (symptom score, oxygen saturation and pulse rate), for each patient. The statistics for each patient and for each data type were determined during a run-in period (6-week or 40 sets of diaries completed), from which the probability density functions (PDF) were estimated for each data type, providing an individualised probability of encountering a specific value for that data type, for that patient. From this information, a cumulative density function was computed and used to set a patient-specific alert threshold (95 % centile), as illustrated in Fig. 1. The data were displayed to the respiratory research nurse using time plots that showed when a patient-specific threshold was exceeded (“alert condition”). However these alerts were not visible to the patients in the cohort study.

To analyse usage patterns, user interactions with the tablet were recorded and data analytics about the use of the software application were synchronised with the remote server. Using this information we computed the time taken to use each separate section of the application, the symptom diary, and the videos. In order to estimate the monthly trend of the time taken to complete the symptom diary and oxygen saturation measurement, a cumulative distribution function was computed from daily data for all patients in each month of the study.

## Results

### Use of mHealth system

Twenty-three patients were given the mHealth system to use for a 6-month period, with 18 of these completing the full six-month study. One patient died and four others did not complete the study: there were technical problems affecting one patient’s use of the tablet on day 13 which, although resolved, led to the patient deciding to withdraw; two patients had multiple hospital admissions and became too unwell and one patient disliked the system describing it as ‘too slow’. Patients were recruited following hospital admission for an exacerbation of COPD, and from respiratory out-patient clinics, pulmonary rehabilitation programmes and GP practices Table 2. Gives details of participant characteristics, they had moderate to severe COPD as defined by MRC dyspnoea score and Gold Classification and were of a wide age range. Participants were prescribed a range of COPD related medication, with the majority of patients using inhaled salbutamol, salmeterol and tiotropium. Participants had a range of pre-existing computer skills from not having a computer to using it regularly. The initial home visit by the respiratory research nurse included time to instruct on tablet use, which took between 10 and 20 min, depending on prior patient experience of using similar technology. Three months after commencing the study, 95 % of diaries and oxygen saturation measurements were completed within 100 s. Moreover, patient did not reported any difficulty with entering their data on the EDGE mobile application [14].

**Table 2:** Patient demographics

	Number (%)
Age	40-50 year 6 %
	51-60 year 6 %
	61-70 year 33 %
	71-80 year 44 %
	81-90 year 11 %
Gender (M;F)	Male 9 (50 %), Female 9 (50 %)
COPD severity GOLD1	0 (0 %)
GOLD 2	6 (33 %)
GOLD 3	9 (50 %)
GOLD 4	2 (11 %)
Not recorded at entry to study	1 (6 %)
Computer skills	83 % had a computer, of whom
	13 % Never used their computer
	13 % Hardly ever used their computer
	20 % Sometimes used their computer
	7 % Quite frequently used their computer
	47 % Used their computer every day
MRC dyspnoea scale	MRC 2 - 0 %,
	MRC 3 - 89 %
	MRC - 4 11 %
Hospital admissions during study	9 hospital admission in total, 7 COPD related, 1 cardiac related and 1 lung surgery

**Fig. 2:**
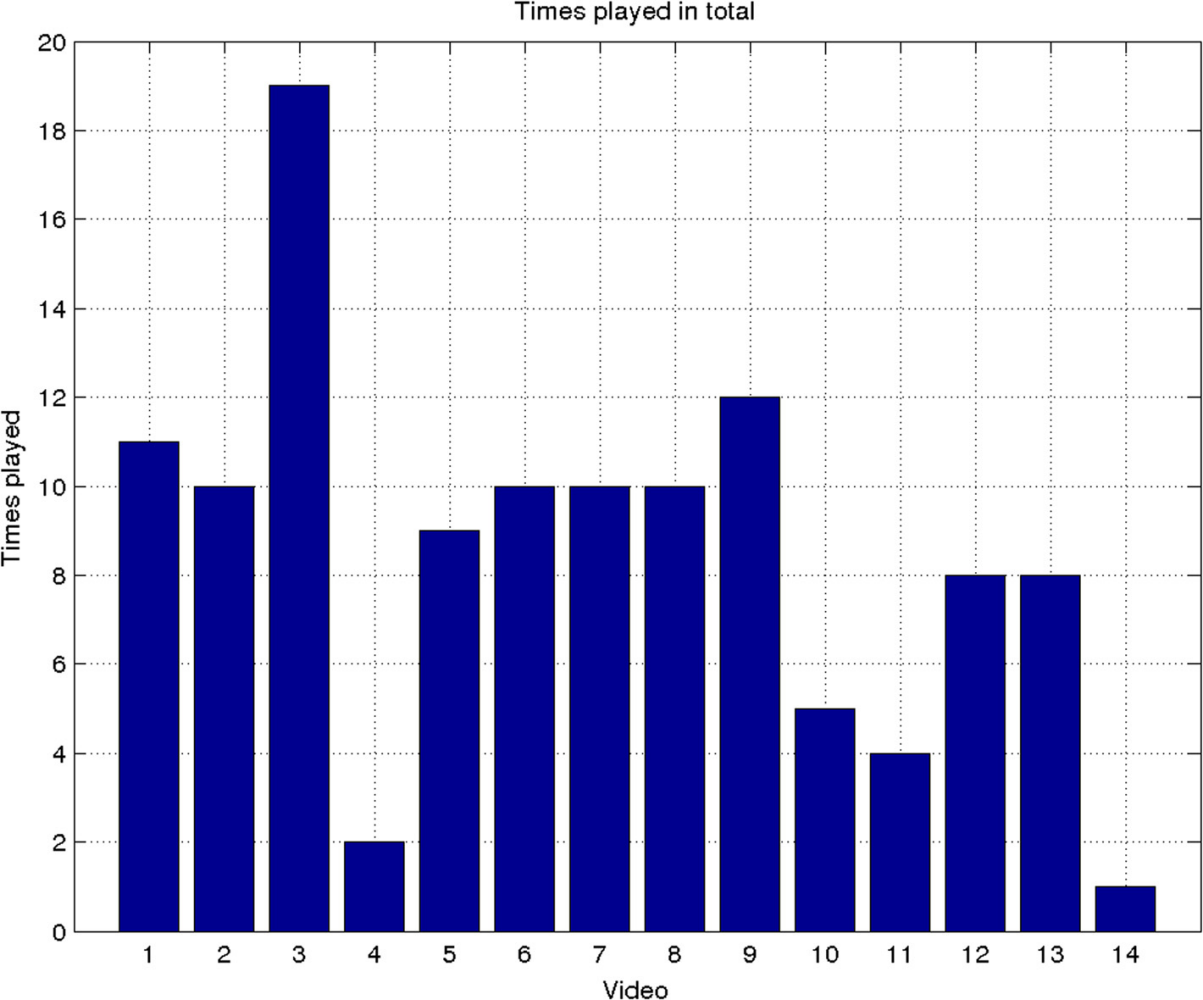
Number of times videos watched. Number of times each video was watched during the cohort study

Patients completed the symptom score and medication use diary, and recorded their oxygen saturation and pulse rate for an average of 77 % of trial days (range 40-98 %), Table 3 presents a summary of the diary scores and vital signs recorded during the cohort study. 78 % of participants completed their diaries and measured their oxygen saturation and pulse rate on at least 5 days/week throughout the six months. Self-management plans were accessed by all patients (mean 9.8 times, range 3–40). 16 of 18 patients accessed the self-management videos (mean 3.9 times, range 0–11). All 14 individual video clips were accessed (mean 8.5 times, range 1–19), the most popular video being “How to use an MDI inhaler”. Video clips about inhaler technique, exercise and pulmonary rehabilitation and mood management were watched a similar number of times (means of 8.1, 9.8 and 7.6 times respectively). 17 patients accessed messages from the respiratory research nurse (mean 29.7 times, range 0–169) (Fig. 2).

**Table 3:** Symptom scores, pulse rate, and Oxygen saturation statistics for run-in and follow-up period

Trait	Run-in mean (std)	Follow-up mean (std)
Symptom scores	9.6 (2.4)	9.1 (2.7)
Pulse rate	83.4 (17.1)	83.5 (16.9)
Oxygen Saturation	93.8 (3.0)	93.2 (3.6)

### Alerts and their relationship to events

There were a total of 189 alerts generated: 47 symptom diary alerts were received from 16 participants (mean 2.9, SD1.6), 80 pulse rate alerts were received from 18 participants (mean 4.4, SD2.5) and 62 oxygen saturation alerts were received from 17 participants, (mean 3.6 SD2.6). 37 ‘exacerbation’ events were identified in 16 participants (mean 2.3, SD 1.5). Of the 37 events, 15 (40.5 %) were preceded by one or more alert in the 3 days prior to the event. Events associated with alerts were identified in 11 (61 %) of the participants. In total, 4 false positives (event flagged that did not correspond to a medication event) and 4 false negatives (medication events that were not anticipated by any alert) were identified.

## Discussion

We have shown that regardless of previous computer experience and age, a well-designed mHealth system can be made intuitive and easy to use. Patients could be taught how to use the tablet in a short dedicated visit, and within an acceptable time frame of three months were entering data regularly at a time of their choosing. Compliance with the mHealth system was high, with the majority of patients using the pulse oximeter to measure their oxygen saturation and pulse rate, and record their symptoms and medicine use on a regular basis. Most accessed the range of self-management support modules available including their self-management plan and the range of video clips, especially those focusing on inhaler technique. Patients also accessed messages from the respiratory research nurse demonstrating the acceptability of this form of communication with a healthcare professional^1^.

The estimation of individualised alert thresholds was feasible, and a proportion of alerts occurred during the three days preceding ‘events’, when patients decided to initiate their standby medicines to treat an exacerbation.

This was a small preliminary study focussing on the usability of the mHealth system and the self-management support modules it included. Patients appeared to like having access to video content to support inhaler technique. This represents a potentially new way of teaching good inhaler technique, which is vital to achieving the clinical benefits of appropriate inhaler use. Videos were accessed in all subject areas and often on multiple occasions demonstrating the flexibility of the system for users. This multi-media format, which can also be used to deliver exercise advice from pulmonary rehabilitation programmes, provides a means of reinforcing self-management strategies. It is possible that patient interest in videos would wane over time, however, and this study was too short to evaluate this.

In contrast to other studies [12, 13], our telehealth intervention included a substantial element of support for self-management. Patients could enter data at a time of their choosing (although the analysis of the application usage showed that patients consistently used the application at about the same time, ± 1 h), and there was an opportunity for them to monitor their data and to observe trends over time. Patients did not voice any concerns that their data was reviewed only on a weekly basis, with a respiratory research nurse using the messaging facility as appropriate, and continued to interact with their usual healthcare professionals as required. Existing telehealth systems have often restricted times of data entry rather than fitted with individual patient lifestyles, with frequent data errors requiring repeated data entry. They have also tended to rely on remote monitoring by clinicians rather than on empowering patients to make their own health care decisions [12, 13, 16]. Fixed alerting thresholds are based on the average experience of a cohort [15, 16]. However, patients can be stable, whilst providing physiological measurements that might permanently lead to alerts (e.g. 92 % is often used as a threshold for alerting on Oxygen saturation, yet some patients are stable at lower levels, and some patients might repeatedly report certain symptoms thus increasing their scores). By producing a user-friendly mHealth system that is capable of flexible integration into existing care pathways, there is now an opportunity for optimising existing care pathways to deliver best healthcare outcomes in a cost-effective fashion. This approach needs to be further evaluated and compared with standard care. The results of this cohort study, along with the results from a qualitative study conducted in parallel with this patient group [14] have been used to inform the design of a randomised controlled trial currently underway.

### Limitations

We used a novel methodology to derive individualised alerting thresholds, and then reviewed medicine use data in the three preceding days to identify any ‘events’ for which patients took their standby medicines to treat an exacerbation. Other than identifying the fact that 40 % of the alerts were related to events, the study was too small to draw firm conclusions about the association between alerts and events, which is likely to be complex and influenced by past patient experience and healthcare beliefs.

The findings of this study need to be validated in a randomised control trial: this is now taking place and recruitment to the 12-month follow-up clinical trial is now complete. The outcomes of this trial will determine whether there are improvements in quality of life with the use of the software application and an associated reduction in use of healthcare resources (primary care clinic attendances and hospital admissions).

The EDGE application was used with a pulse oximeter, in case of co-morbidities the number of external devices used in combination with the application might increase. It is worth noticing that the number of external device might increase the burden to the user, and eventually generate lower compliance rates. For this reason the authors consider very important to experiment the set-up of such interventions with cohort studies like the one described in this article.

## Conclusions

The findings of this cohort study confirm that daily use of the mHealth platform is feasible and acceptable to people with COPD for reporting daily symptoms and medicine use, and to measure physiological variables such as pulse rate and oxygen saturation. Features to support self-management such as video clips, self-management plans and respiratory nursing messaging were accessed by the majority of patients. The development of individualised alert thresholds has the potential to identify exacerbations early but requires further evaluation.

## Figures and Tables

**Fig. 3:**
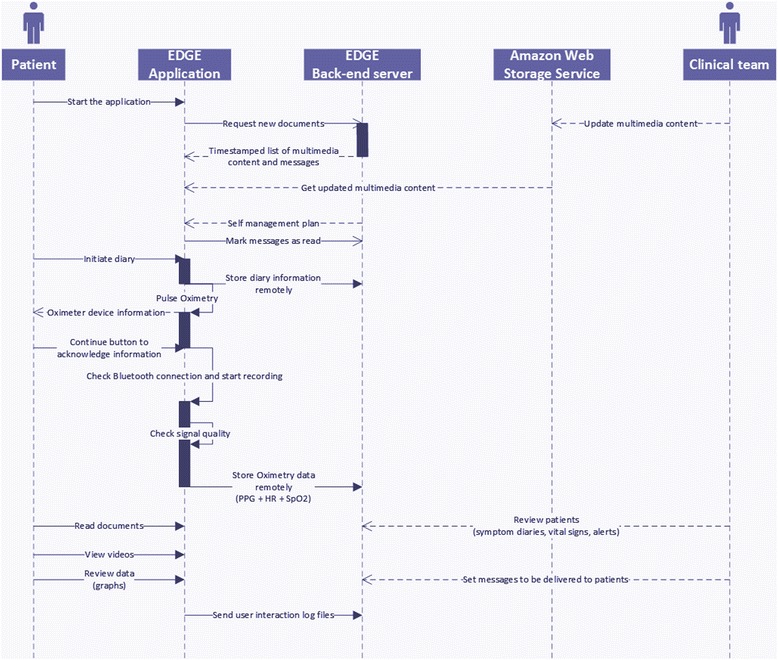
UML interaction diagram

**Fig. 4:**
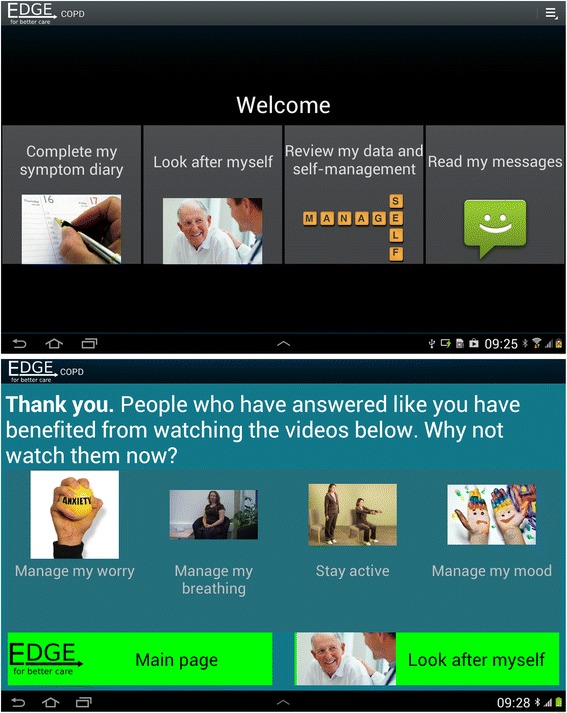
Mobile application user interface

**Fig. 5:**
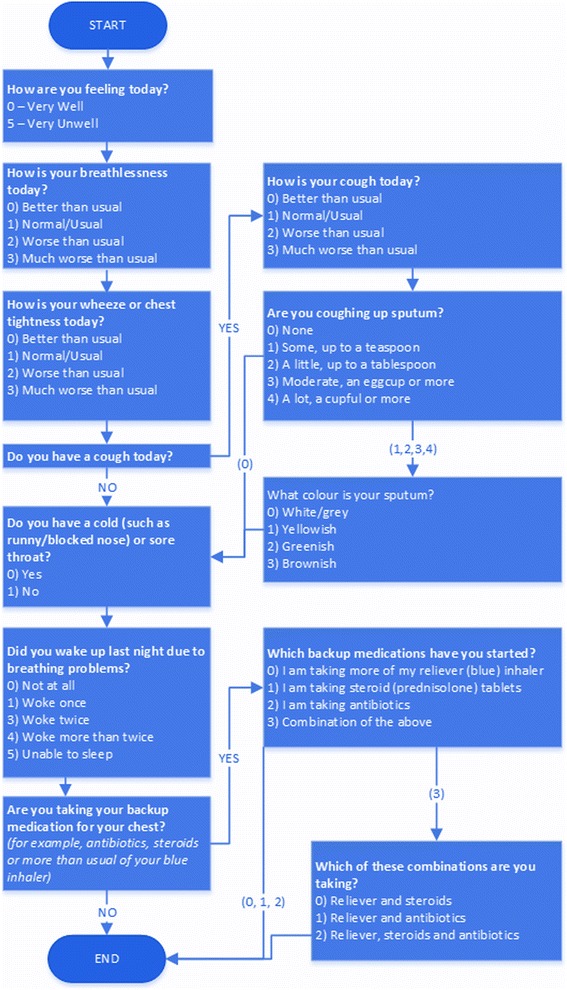
Daily symptom questionnaire

## Appendix

### Schema of interactions

Figure 3 displays the interactions between the actors (patients and health specialists) and the EDGE m-Health system.

### m-Health application

Figure 4 shows the application user interface. The design strategy focussed on usability and readability of the content. A co-design workshop was employed to collect feedback from a group of patients.

### Questionnaires

Here we report the set of questions used in the cohort study. The flowchart shows the flow of questions designed such that the patients will be asked only pertinent questions (Fig. 5).

## Footnotes

1 We assessed patient acceptance using qualitative methods, the results of which have been published in a separate paper [14].
